# Real-world experience of first-line afatinib in patients with *EGFR*-mutant advanced NSCLC: a multicenter observational study

**DOI:** 10.1186/s12885-019-6107-1

**Published:** 2019-09-09

**Authors:** Gwo-Fuang Ho, Chee-Shee Chai, Adlinda Alip, Mohd Ibrahim A. Wahid, Matin Mellor Abdullah, Yoke-Ching Foo, Soon-Hin How, Adel Zaatar, Kai-Seng Lam, Kin-Wah Leong, John-Seng-Hooi Low, Mastura Md Yusof, Erica Chai-Yong Lee, Yok-Yong Toh, Chong-Kin Liam

**Affiliations:** 1 Department of Clinical Oncology, Faculty of Medicine, University of Malaya, Kuala Lumpur, Malaysia; 2 Department of Medicine, Faculty of Medicine and Health Science, University Malaysia Sarawak, Kota Samarahan, Sarawak Malaysia; 3 Beacon International Specialist Centre, Kuala Lumpur, Malaysia; 4 Subang Jaya Medical Centre, Kuala Lumpur, Malaysia; 5 Hospital Tengku Ampuan Afzan, Kuantan, Malaysia; 6 Gleneagles Hospital, Penang, Malaysia; 7 Pantai Hospital, Kuala Lumpur, Malaysia; 8 Department of Medicine, Faculty of Medicine, University of Malaya, Kuala Lumpur, Malaysia

**Keywords:** Afatinib, Dose adjustment, E*pidermal growth factor receptor* (*EGFR*), Real-world, Tyrosine kinase inhibitor

## Abstract

**Background:**

This study aimed to evaluate the efficacy, side-effects and resistance mechanisms of first-line afatinib in a real-world setting.

**Methods:**

This is a multicenter observational study of first-line afatinib in Malaysian patients with epidermal growth factor receptor (*EGFR*)*-*mutant advanced non-small cell lung cancer (NSCLC). Patients’ demographic, clinical and treatment data, as well as resistance mechanisms to afatinib were retrospectively captured. The statistical methods included Chi-squared test and independent t-test for variables, Kaplan-Meier curve and log-rank test for survival, and Cox regression model for multivariate analysis.

**Results:**

Eighty-five patients on first-line afatinib from 1st October 2014 to 30th April 2018 were eligible for the study. *EGFR* mutations detected in tumors included *exon 19* deletion in 80.0%, *exon 21 L858R* point mutation in 12.9%, and rare or complex *EGFR* mutations in 7.1% of patients. Among these patients, 18.8% had Eastern Cooperative Oncology Group performance status of 2–4, 29.4% had symptomatic brain metastases and 17.6% had abnormal organ function.

Afatinib 40 mg or 30 mg once daily were the most common starting and maintenance doses. Only one-tenth of patients experienced severe side-effects with none having grade 4 toxicities. The objective response rate was 76.5% while the disease control rate was 95.3%. At the time of analysis, 56 (65.9%) patients had progression of disease (PD) with a median progression-free survival (mPFS) of 14.2 months (95% CI, 11.85–16.55 months). Only 12.5% of the progressed patients developed new symptomatic brain metastases. The overall survival (OS) data was not mature. Thirty-three (38.8%) patients had died with a median OS of 28.9 months (95% CI, 19.82–37.99 months). The median follow-up period for the survivors was 20.0 months (95% CI, 17.49–22.51 months).

Of patients with PD while on afatinib, 55.3% were investigated for resistance mechanisms with *exon 20 T790 M* mutation detected in 42.0% of them.

**Conclusions:**

Afatinib is an effective first-line treatment for patients with *EGFR*-mutant advanced NSCLC with a good response rate and long survival, even in patients with unfavorable clinical characteristics. The side-effects of afatinib were manageable and *T790 M* mutation was the most common resistance mechanism causing treatment failure.

## Background

*Epidermal growth factor receptor* (*EGFR*)-tyrosine kinase inhibitor (TKI) is the recommended first-line treatment for patients with advanced non-small cell lung cancer (NSCLC) harboring somatic driver mutation in the *EGFR* gene [1]. Several phase III clinical trials have reported promising median progression-free survivals (mPFS) (9–13 months) and tolerable side-effects in patients with *EGFR*-mutant advanced NSCLC receiving first-generation *EGFR*-TKIs [2–6].

Afatinib is an irreversible, second-generation *EGFR*-TKI that has been shown to be more potent than platinum doublet chemotherapies as well as the first-generation *EGFR*-TKIs, such as gefitinib and erlotinib [7–10]. In the LUX-Lung 7 study, patients receiving first-line afatinib for *EGFR* mutant advanced NSCLC had significantly longer mPFS and median time-to-treatment failure than those on first-line gefitinib [9]. In LUX-Lung 8, patients receiving second-line afatinib for advanced squamous cell carcinoma of lung had significantly longer mPFS and median overall survival (mOS) than those on second-line erlotinib [10]. Since afatinib targets all homo-dimers and hetero-dimers of the ErbB family (*EGFR*/ErbB1, *HER2*/ErbB2, ErbB3, and ErbB4), it is more efficacious than first-generation *EGFR*-TKIs [11, 12]. At the same time, the broad spectrum of activity and irreversible mechanism of action of afatinib also lead to more treatment related side-effects.

Patients with rare or complex *EGFR* mutation, symptomatic brain metastases, poor Eastern Cooperative Oncology Group (ECOG) performance status and inadequate organ function are routinely excluded from clinical trials. Nevertheless, these unfavourable characteristics are commonly encountered in clinical practice. Therefore, this study aimed to look into the efficacy and side-effects of first-line afatinib in the real-world setting. In addition, the mechanisms of acquired resistance causing first-line afatinib failure were analyzed.

## Methods

### Study design and patients

This is a multicenter observational study of Malaysian patients with *EGFR-*mutant advanced NSCLC started on first-line afatinib treatment at the University of Malaya Medical Center, Subang Jaya Medical Center, Beacon International Specialist Hospital, Pantai Hospital Kuala Lumpur, Gleneagles Hospital Penang and Hospital Tengku Ampuan Afzan Kuantan from 1st October 2014 to 30th April 2018. All patients analyzed were aged 18 years and above, had histologically confirmed locally advanced (stage IIIB) or metastatic (stage IV) NSCLC and had *EGFR* mutation detected in the pre-treatment biopsy specimens. Patients were excluded if they had previous cytotoxic chemotherapy or targeted therapy. Patients with symptomatic brain metastases and inadequate organ function were not excluded. The study was approved by the ethics committees of the respective hospitals that also granted an informed consent waiver.

### Procedure

Eligible patients were retrospectively identified from the lung cancer databases and pharmacy dispensing records of the respective hospitals. The patients’ demographic, clinical, and treatment data, as well as resistance mechanisms to afatinib were extracted from their case records. A never smoker was defined as one with lifetime cigarette smoking of less than 100 sticks [13]. The patients’ organ function at diagnosis was graded according to Common Terminology Criteria for Adverse Events version 4 (CTCAE v4.0) for blood, renal and liver function [14]. Initial tumor biopsy specimens of the patients were tested for *EGFR* mutations using the cobas® *EGFR* Mutation Test v2 (Roche Molecular Systems, New Jersey, USA), or peptic nucleic acid-locked nucleic acid polymerase chain reaction (PCR) clamp method, PNAClamp™ *EGFR* Mutation Detection Kit (PANAGEN, Daejon, Korea). Baseline computed tomography (CT) examination of the thorax, abdomen and pelvis (TAP) was performed in every patient at diagnosis. CT-brain was performed in those with neurological symptoms or signs. The patient’s NSCLC was staged according to the 7th edition of the American Joint Committee on Cancer [15]. Tumor response was evaluated by performing a repeat CT-TAP 4 weeks after the initiation of afatinib, and subsequently, once every 12 weeks until disease progression or symptomatic deterioration, whichever occurred earlier. Tumor response was categorized according to the Response Evaluation Criteria in Solid Tumors version 1.1 [16].

Patients received afatinib at starting doses of 40 mg, 30 mg, 25 mg or 20 mg once daily. Afatinib 40 mg once daily is the recommended starting dose. Afatinib at 30 mg once daily was only started in patients with *exon 19* deletion or *exon 21 L858R* point mutation who did not have symptomatic brain metastases. Afatinib 20 mg once daily and 25 mg once daily were derived by dividing the 40 mg and 50 mg tablets into halves, respectively. These adjusted dosages were only given to patients who were financially constrained to self-purchase the drug. The maintenance dose of afatinib ranged from 20 to 50 mg once daily depending on the patients’ clinical response and tolerability. The optimum dose of afatinib was defined as the dose that could control the patient’s disease alongside tolerable side-effects for the patient. Afatinib was given until symptomatic disease progression or occurrence of intolerable side-effects. Only common side-effects documented during clinic visits such as diarrhea, stomatitis, skin rash, acne, paronychia and fatigue were assessed and graded according to CTCAE v4.0 [14]. Second-line treatment was offered when patients experienced symptomatic disease progression confirmed by CT scan or intolerable side-effects from afatinib. At any time, patients with symptomatic brain metastases were offered surgical resection, whole brain radiotherapy or stereotactic radiotherapy for brain lesions based on the decision of the multidisciplinary team in the respective centers.

Investigations for acquired *exon 20 T790 M* mutation and histological transformation were only performed in patients who had PD after 31st December 2015 when early access to the third-generation *EGFR*-TKI, osimertinib became available. Investigation for *T790 M* mutation involved tissue re-biopsy or liquid biopsy. The former utilized the similar *EGFR* mutation detection technique as at initial diagnosis; while for the latter peptic nucleic acid-locked nucleic acid polymerase chain reaction (PCR) clamp method (PANAGEN, Daejon, Korea) or p-*EGFR* droplet digital PCR-based technology (Sanomics, Hong Kong, China) was used.

### Statistical analysis

Categorical variables were expressed as percentages while continuous variables were expressed as mean ± standard deviation (SD) or median with range depending on the normality of distribution of the variables. Kaplan-Meier methodology was used to determine the mPFS and mOS. Differences between categorical variables were tested using Chi-Squared test or Fisher Exact test. For continuous variables, the differences were compared using independent t-test or Mann-Whitney U test. Multivariate analysis was performed using logistic regression. A p-value of < 0.05 was considered statistically significant. Statistical analyses were performed by using the software package, Statistical Package for the Social Sciences (SPSS for Windows version 23.0, SPSS Inc., Chicago, IL, USA).

## Results

### Demographic and clinical characteristics

**Fig. 1:**
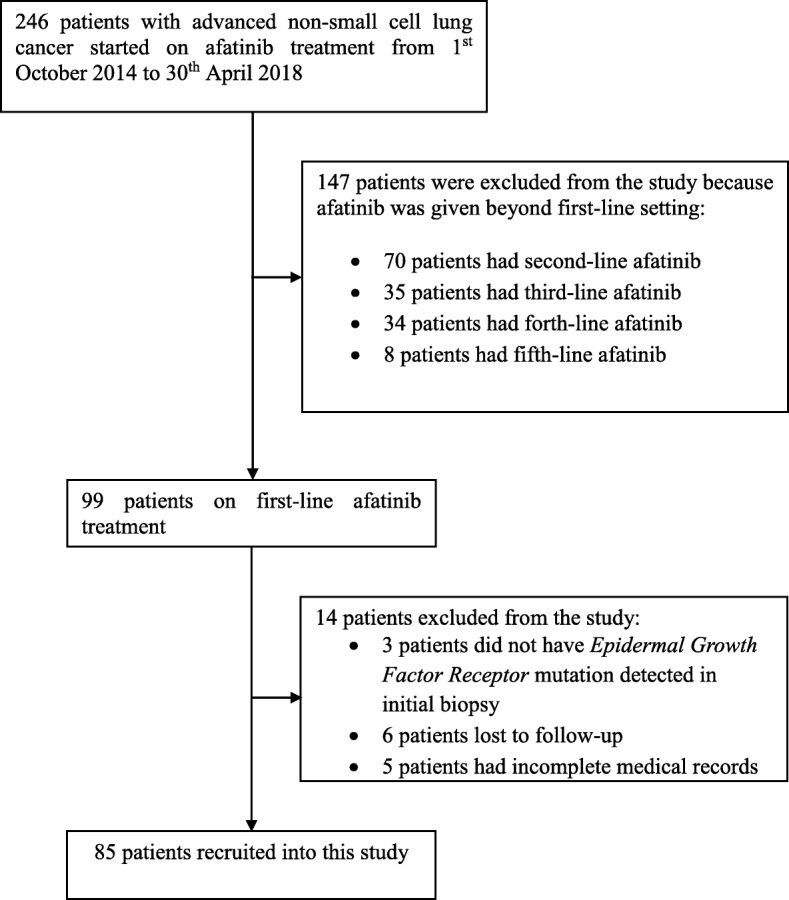
Flow of patient selection according to inclusion criteria

A total of 85 patients who met the study criteria were included (Fig. 1). Their demographic and clinical characteristics are shown in Table 1. The majority of the patients were female, never smokers and of Chinese ethnicity. Eighty-two (96.5%) patients had lung adenocarcinoma while the remaining had squamous cell carcinoma. The *EGFR* mutations harbored by the tumors included *exon 19* deletion in 80.0%, *exon 21 L858R* point mutation in 12.9%, and rare or complex *EGFR* mutations in 7.1% of the patients. The ECOG performance status was 2–4 in 18.8%, symptomatic baseline brain metastases were present in 29.4%, and abnormal organ function at baseline was present in 17.6% of the patients.

**Table 1:** Demographic and clinical characteristics of patients

Demographic and clinical characteristic	No. of patients (*n* = 85)
Age, year	
Mean (+ SD)	59.1 + 10.8
Gender, No. (%)	
Female	47 (55.3)
Male	38 (44.7)
Ethnicity, No. (%)	
Chinese	63 (74.1)
Non-Chinese (Malay and Indian)	22 (25.9)
Smoking history, No. (%)	
Never smoker	67 (78.8)
Previous or current smoker	18 (21.2)
ECOG performance status at diagnosis, No. (%)	
ECOG 0–1	69 (81.2)
ECOG 2–4	16 (18.8)
Tumor histology, No. (%)	
Adenocarcinoma	82 (96.5)
Squamous cell carcinoma	3 (3.5)
Tumor stage, No. (%)	
IIIB	4 (4.7)
IV	81 (95.3)
Symptomatic baseline brain metastases, No. (%)	
No	60 (70.6)
Yes	25 (29.4)
-Parenchymal metastases	16 (64.0)
-Leptomeningeal metastases	7 (28.0)
-Both parenchymal and leptomeningeal metastases	2 (8.0)
Abnormal organ function, No. (%)	
No	70 (82.4)
Yes	15 (17.6)
-Blood	5 (33.3)
-Renal	9 (60.0)
-Liver	2 (13.3)
*EGFR* mutation subtype, No. (%)	
*Exon 19* deletion	68 (80.0)
E*xon 21 L858R* point mutation	11 (12.9)
Rare or complex mutations	6 (7.1)
-*Exon 18 G719X*	2 (33.1)
-*Exon 18 G71*9X and *exon 20 S768I*	1 (16.7)
-*Exon 18 G719X* and *exon 20 T790 M*	1 (16.7)
-*Exon 19* deletion and *exon 20 insertion*	1 (16.7)
-*Exon 20* insertion	1 (16.7)

*Abbreviations*: *ECOG* Eastern Cooperative Oncology Group, *EGFR epidermal growth factor receptor*

### Afatinib starting dose, dose adjustment and optimal dose and treatment of baseline brain metastases

Most of the patients were started on afatinib 40 mg once daily (52.9%), followed by 30 mg once daily (35.3%), 20 mg once daily (8.2%) and 25 mg once daily (3.5%) (Table 2). The initial starting dose of afatinib could be maintained in more than half of the patients. Afatinib dose reduction was exclusively due to side-effects while dose escalation was because of inadequate treatment response. The optimum dose of afatinib was 40 mg once daily or 30 mg once daily in 35.7 and 35.7% of the patients, respectively. Of the 25 patients with baseline symptomatic brain metastases, 21 (84.0%) had brain radiotherapy or surgical resection of the brain lesions on top of the first-line afatinib (Table 2).

**Table 2:** Afatinib starting dose, dose adjustment and optimal dose and treatment of baseline brain metastases

Treatment Pattern and Outcome	Total number of patients (*n* = 85)
Afatinib starting dose, No. (%)	
40 mg once daily	45 (52.9)
30 mg once daily	30 (35.3)
25 mg once daily	3 (3.5)
20 mg once daily	7 (8.2)
Afatinib dose adjustment, No. (%)	
Starting dose maintained	49 (57.6)
Dose increased	10 (11.8)
Dose reduced	26 (30.6)
Afatinib optimum dose, No. (%)	
50 mg once daily	4 (4.7)
40 mg once daily	30 (35.3)
30 mg once daily	30 (35.3)
25 mg once daily	12 (14.1)
20 mg once daily	9 (10.6)
Brain metastasis treatment, No. (%)	
No brain metastases	60 (70.6)
Afatinib alone	4 (4.7)
Afatinib with surgery or radiotherapy	21 (24.7)

### Treatment outcome

#### Response to afatinib

The objective response rate (ORR) was 76.5% while the disease control rate (DCR) was 95.3% on first-line afatinib (Table 3). Two (2.4%) patients had complete response. The ORR and DCR according to *EGFR* mutation subtype, presence or absence of symptomatic brain metastases, ECOG performance status, presence or absence of abnormal organ function, afatinib dose adjustment and different optimal doses of afatinib are shown in Table 4. Patients without baseline symptomatic brain metastases had significantly better response to afatinib than those with symptomatic baseline brain metastases (81.7 versus 56.0%, *p* = 0014). On multivariate subgroup analyses involving the covariates as shown in Table 4, patients without symptomatic brain metastases had significantly higher ORR than that of those with symptomatic brain metastases (81.7 versus 56.0%; OR, 4.51; 95% CI, 1.45–14.00; p = 0.009); while patients with afatinib dose reduction had significantly higher ORR than that of those without dose adjustment (88.5 versus 65.3%, OR, 5.53; 95% CI, 1.32–23.24; *p* = 0.019).

**Table 3:** Treatment outcome to afatinib and resistance mechanism identified at disease progression

Treatment outcome	Total number of patients (*n* = 85)
Best tumor response, No. (%)	
Complete response	2 (2.4)
Partial response	63 (74.1)
Stable disease	16 (18.8)
Progressive disease	4 (4.7)
Disease progression site, No. (%)	
None	29 (34.1)
New brain lesions	7 (8.2)
New lesions at other sites	49 (57.6)
Investigation for resistance mechanism, No. (%)	
No progression	29 (34.1)
Not investigated	25 (29.4)
Investigated	31 (36.5)
-*Exon 20 T790 M* mutation detected	13 (42.0)
-*Exon 20 T790 M* mutation not detected and no histologic transformation	18 (58.0)

**Table 4:** Univariate and multivariate analyses of ORR and DCR according to clinical and treatment characteristics

Characteristics	ORR, No. (%)	^*^p-value	^g^OR (95% CI), *p*-value	DCR, No. (%)	^*^p-value	^g^OR (95% CI), *p*-value
*EGFR* mutation subtype, No. (%)						
*Exon 19* deletion	52 (76.5)	0.265	2.27 (0.47–11.01), 0.309^a^	64 (94.1)	0.263	2.72 (0.41–18.24), 0.302^a^
*Exon 21 L858R* point mutation	6 (54.5)		0.40 (0.03–5.21), 0.485^b^	9 (81.8)		2.28 (0.31–16.62), 0.420^b^
Rare and complex mutation	5 (83.3)			6 (100)		
Baseline symptomatic brain metastases, No. (%)						
No	49 (81.7)	0.014	4.51 (1.45–14.00), 0.009^#^	57 (95.0)	0.251	3.0 (0.55–16.38), 0.205^#^
Yes	14 (56.0)			22 (88.0)		
ECOG performance status, No. (%)						
0–1	49 (71.0)	0.175	0.27 (0.05–1.44), 0.125^#^	64 (92.8)	0.889	0.79 (0.08–8.76), 0.835^#^
2–4	14 (87.5)			15 (93.8)		
Abnormal organ function, No. (%)						
No	53 (75.7)	0.468	1.27 (0.28–5.81), 0.755^#^	64 (91.4)	0.585	0.57 (0.07–4.97), 0.616 ^#^
Yes	10 (66.7)			15 (100)		
Afatinib dose adjustment, No. (%)						
Dose reduced	23 (88.5)	0.084	5.53 (1.32–23.24), 0.019^c^	25 (96.2)	0.729	3.22 (0.29–35.40), 0.339^c^
Dose increased	8 (80.0)		2.13 (0.36–12.57), 0.404^d^	9 (90.0)		1.30 (0.11–15.02), 0.835^d^
Starting dose maintained	32 (65.3)			45 (91.8)		
Optimal afatinib dose, No. (%)						
Less than 40 mg once daily	40 (78.4)	0.156	2.03 (0.59–6.94), 0.259^e^	47 (92.2)	0.836	0.88; 0.13–6.13, 0.895^e^
40 mg once daily	19 (63.3)			28 (29.3)		
50 mg once daily	4 (100)^f^			4 (100)^f^		

*Abbreviations*: *ORR* objective response rate, *DCR* disease control rate, *OR* odd ratio, *95% CI* 95% confidence interval, *EGFR epidermal growth factor receptor, ECOG* Eastern Cooperative Oncology Group

*p-value of Chi-square test

^#^second parameter was the reference group

^a^*exon 19* deletion versus *exon 21 L858R* point mutation; ^b^*exon 19* deletion versus rare and complex mutations

^c^afatinib dose reduced versus starting dose maintained; ^d^afatinib dose increased versus starting dose maintained

^e^afatinib less than 40 mg once daily versus 40 mg once daily

^f^afatinib dose 50 mg once daily not compared because of the small number of patients

^g^multivariate analysis with cox regression

#### Progression-free survival

**Fig. 2:**
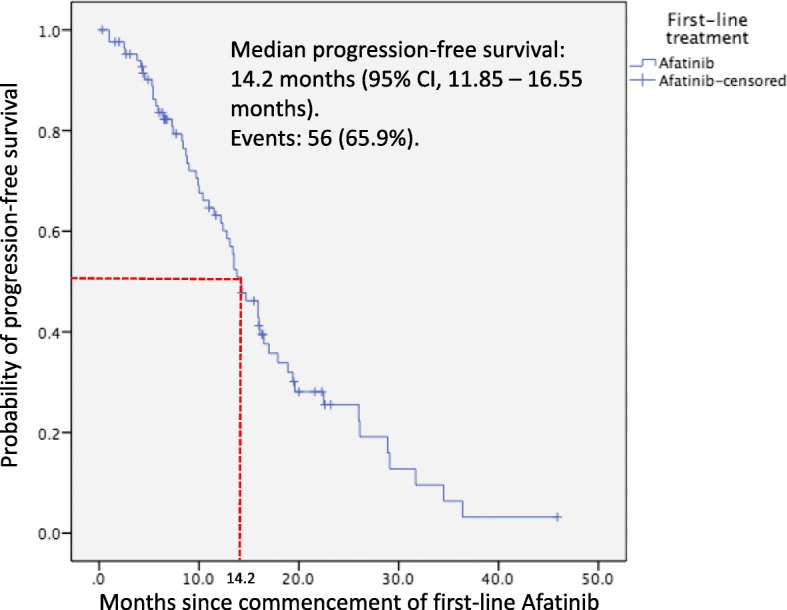
Kaplan-Meir plot for progression-free survival of patients on first-line afatinib

The mPFS was 14.2 months (95% CI, 11.85–16.55 months) with 56 (65.9%) patients having PD at the time of analysis (Fig. 2). Only 12.5% of patients with PD experienced new symptomatic brain metastases while the remaining had PD at new sites other than the brain. The mPFS according to *EGFR* mutation subtype, presence or absence of symptomatic brain metastases, ECOG performance status, presence or absence of abnormal organ function, afatinib dose adjustment and different optimal doses of afatinib are shown in Table 5. On univariate analysis, only patients with *exon 19* deletion had significantly longer mPFS compared to patients with *exon 21 L858R* point mutation (16.0 versus 8.7 months; HR, 0.31; 95% CI, 0.14–0.71; p = 0.006) and rare or complex *EGFR* mutations (16.0 versus 9.0 months; HR, 0.34; 95% CI, 0.13–0.94, *p* = 0.037). On multivariate analysis, only the mPFS of patients with *exon 19* deletion was significantly longer than the mPFS of patients with *exon 21 L858R* point mutation (16.0 versus 8.7 months; HR, 0.27; 95% CI, 0.12–0.58; *p* = 0.001).

**Table 5:** Univariate and multivariate analyses of progression-free survival according to clinical and treatment characteristics

Characteristics	Patients, No. (%)	mPFS (months)	Univariate analysis		Multivariate analysis	
			HR (95% CI)	*p*-value	HR (95% CI)	*p*-value
*EGFR* mutation subtype, No. (%)						
*Exon 19* deletion	68 (80.0)	16.0	0.31 (0.14–0.71)^a^	0.006	0.27 (0.12–0.58)^a^	0.001
*Exon 21 L858R* point mutation	11 (12.9)	8.7	0.34 (0.13–0.94)^b^	0.037	0.39 (0.15–1.03)^b^	0.058
Rare and complex mutation	6 (7.1)	9.0				
Baseline symptomatic brain metastases, No. (%)						
No	60 (70.6)	14.3	0.67 (0.34–1.27)^g^	0.209	0.70 (0.37–1.32)^g^	0.267
Yes	25 (29.4)	13.5				
ECOG performance status, No. (%)						
0–1	69 (81.2)	13.8	0.86 (0.39–1.90)^g^	0.703	0.86 (0.39–1.90)^g^	0.703
2–4	16 (18.8)	15.9				
Abnormal organ function, No. (%)						
No	70 (82.4)	14.3	0.53 (0.25–1.09)^g^	0.086	0.50 (0.25–1.00)^g^	0.050
Yes	15 (17.6)	8.8				
Afatinib dose adjustment, No. (%)						
Dose reduced	26 (30.6)	15.9	0.93 (0.44–1.99)^c^	0.854	0.72 (0.39–1.34)^c^	0.301
Dose increased	10 (11.8)	13.5	2.35 (0.86–6.47)^d^	0.098	2.13 (0.93–4.88)^d^	0.075
Starting dose maintained	49 (57.6)	13.4				
Optimal afatinib dose, No. (%)						
Less than 40 mg once daily	51 (60.0)	15.9	0.64 (0.32–1.28)^e^	0.209	0.47 (0.21–1.08)^e^	0.075
50 mg once daily	4 (4.7)	13.5	0.97 (0.25–3.78)^f^	0.962	1.03 (0.27–4.01)^f^	0.962
40 mg once daily	30 (35.3)	13.4				

*Abbreviations*: *PFS* progression-free survival, *mPFS* median PFS, *HR* hazard ratio, *95% CI* 95% confidence interval, *EGFR epidermal growth factor receptor, ECOG* Eastern Cooperative Oncology Group

^a^*exon 19* deletion versus *exon 21 L858R* point mutation; ^b^*exon 19* deletion versus rare and complex mutations

^c^afatinib dose reduced versus starting dose maintained; ^d^afatinib dose increased versus starting dose maintained

^e^afatinib less than 40 mg once daily versus 40 mg once daily; ^f^afatinib 50 mg once daily versus 40 mg once daily

^g^the second group was the reference category in logistic regression analysis

#### Overall survival

**Fig. 3:**
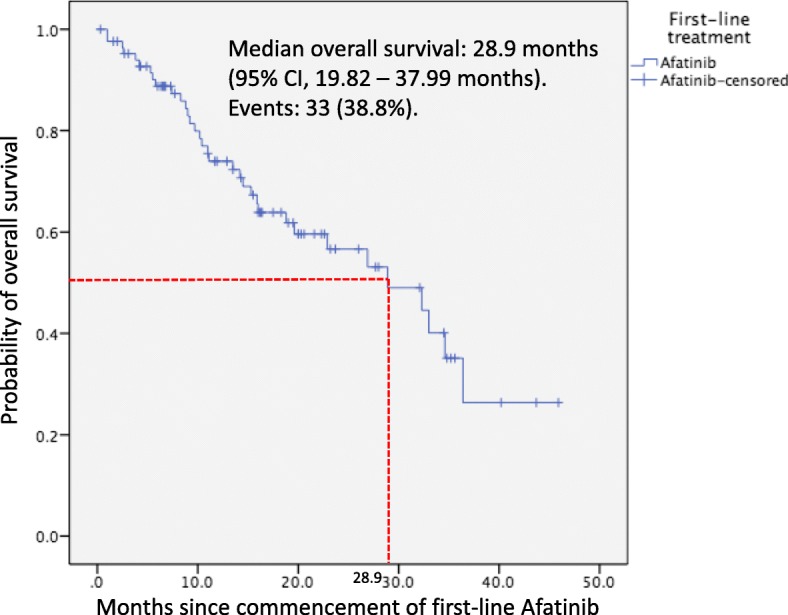
Kaplan-Meir plot for overall survival of patients on first-line afatinib

The mOS was 28.9 months (95% CI, 19.82–37.99 months) (Fig. 3). Thirty-three (38.8%) patients had died at the time of analysis while the median follow-up period for the survivors was 20.0 months (95% CI, 17.49–22.51 months).

### Resistance to afatinib

Of 56 patients who experienced PD while on afatinib, only 31 (55.4%) had PD after 31st December 2015 and were investigated for resistance mechanisms (Table 3). *Exon 20 T790 M* mutation was detected in 42.0% of the 31 patients, while no resistance mechanism could be identified in the remaining 58.0%. *T790 M* mutation was detected exclusively in lung adenocarcinoma and was more frequent in female patients (47.1% versus 35.7%, *p* = 0.524).

### Side-effects of afatinib treatment

One-fifth of the patients did not experience any side-effect; while one-tenth of patients experienced severe side-effects while taking afatinib (Table 6). None of the patients had grade 4 side-effects. Acne (70.6%) was the most common side-effect, followed by diarrhea (54.1%), paronychia (40.0%), stomatitis (27.1%) and fatigue (16.5%).

**Table 6:** Side-effects of first-line afatinib

CTCAE grade	Grade 0	Grade 1	Grade 2	Grade 3	Grade 4
Diarrhea, No. (%)	39 (45.9)	25 (29.4)	17 (20.0)	4 (4.7)	0
Stomatitis, No. (%)	62 (72.9)	13 (15.3)	8 (9.4)	2 (2.4)	0
Acne/rash, No. (%)	25 (29.4)	35 (41.2)	20 (23.5)	5 (5.9)	0
Paronychia, No. (%)	51 (60.0)	23 (27.1)	8 (9.4)	3 (3.5)	0
Fatigue, No. (%)	71 (83.5)	13 (15.3)	1 (1.2)	0	0
Side-effects, No (%)	17 (20.0)	59 (69.4)		9 (10.6)	

*Abbreviations*: *CTCAE* Common Terminology Criteria for Adverse Events

## Discussion

In this study, patients with *exon 19* deletion had significantly longer mPFS than those with *exon 21 L858R* point mutation. Most of the patients with rare or complex *EGFR* mutations demonstrated response to afatinib despite a shorter PFS than that of those with *exon 19* deletion. On the other hand, patients with baseline symptomatic brain metastases did not have significantly shorter PFS compared to those without baseline symptomatic brain metastases despite their lower response rate to afatinib. Other unfavorable clinical characteristics frequently encountered in real-world practice such as poor ECOG performance status or abnormal organ function did not significantly affect the response rate to afatinib or PFS, which implies that afatinib works well even in these patients. Afatinib 40 or 30 mg once daily seems to be the optimal maintenance dose which is effective for Malaysian patients and are uncommonly associated with severe side-effects. The need for dose reduction due to side-effects and the ability of the reduced dose to control the disease are reassuring to the treating clinicians. Symptomatic brain metastases causing failure to first-line afatinib were uncommon and acquired *T790 M* mutation is the most common identified resistance mechanism.

The demographic characteristics of our patients were consistent with previous reports, in which females, never smokers and Asians of Chinese ethnicity were predominant [17–19]. The majority of our patients harbored *exon 19* deletion. This could have been due to selection bias whereby the treating clinicians were influenced by the mOS result of the LUX-Lung 3 and LUX-Lung 6 studies which favored first-line afatinib over cytotoxic chemotherapy among patients with *exon 19* deletion [7, 8, 20]. The mPFS and ORR of patients receiving first-line afatinib in the present study correspond to that reported in randomized control trials (RCTs) (11.0–11.1 months; 56.0–70.0%) and other real-world studies (11.8–11.9 months; 67.2–78.4%) [7–9, 21–24]. Another two real-world studies by Wu et al. [25] and Kim et al. [26] however, reported a much longer mPFS (21.0 and 19.1 months, respectively) among their patients receiving first-line afatinib. The former study included 14 patients who achieved a partial response or at least 6 months of stable disease when on first-line afatinib while the latter study only involved patients with ECOG 0–2 which could have contributed to the longer mPFS. Similar to the present study, Liang et al. [21], Tan et al. [22] Kim et al. [26] and Tanaka et al. [24] also consistently highlighted a longer mPFS and better ORR in patients with tumors harboring *exon 19* deletion treated with first-line afatinib compared to those with *exon 21 L858R* point mutation. In patients with complex or rare *EGFR* mutations treated with first-line afatinib, the present study and another three real-world studies reported a modest mPFS and ORR [21, 22, 27]. Similar beneficial response was not seen in such patients treated with first-generation *EGFR*-TKIs [27]. Contrary to the findings by Tan et al. [22], the present study did not find a significantly shorter mPFS among patients with symptomatic brain metastases receiving first-line afatinib [22]. This favorable outcome could be explained by the uniform afatinib starting dose of 40 mg once daily and the comprehensive brain surgery or radiotherapy approach in the present study cohort. On the other hand, the findings of no difference in the survival and response rate among patients without symptomatic brain metastases when given afatinib 40 mg or less than 40 mg once daily in other studies are also in agreement with the present study [21, 23]. In a recent study by Hochmair et al. [28], *exon 19* deletion, absence of active brain metastases and good ECOG performance status were shown to be associated with longer initial and post-progression treatment duration in a cohort of patients who developed *T790 M* mutation following first-line afatinib treatment and subsequently treated with osimertinib. The median treatment duration for subgroups of patients with active brain metastases or poor ECOG performance status on first-line afatinib was 10.4 months in that study.

The present study and other real-world studies report a much lower incidence of grade 3 or 4 afatinib side-effects when compared to the incidence of 36.0–57.0% reported by RCTs [7–9, 21–23, 26]. This could have been due to the lower afatinib starting dose among patients without symptomatic brain metastases and rare or complex *EGFR* mutations in real-world studies. Early dose de-escalation in some patients before developing grade 3 side-effects in real-world practice could be another explanation. Nevertheless, the retrospective nature of these real-world studies could be a confounding factor for under reporting of drug side-effects. Upon PD on first-line afatinib, the incidence of new brain metastases in the present study was lower than that reported by Liang et al. [21] and Campo et al. [29] (18.6–19.0%). The incidence of acquired *T790 M* mutation was comparable to that reported in the literature (32.1–47.6%) but less than that reported in studies involving first-generation *EGFR*-TKIs (49.0–63.0%) [21, 24–26, 30–33].

This study is among the very few real-world analyses that include patients with unfavorable characteristics such as rare or complex *EGFR* mutations, symptomatic brain metastases, poor ECOG performance status and inadequate organ function. These characteristics have been routinely excluded in RCTs but are common challenges in the real-world. The result of our study therefore further complements the existing information on afatinib from RCTs. Another strength of our study is that we attempted to explore the efficacy of afatinib in various doses and highlight the non-inferior response among patients with symptomatic brain metastases on afatinib 40 mg once daily.

This study has several limitations. Its retrospective nature might have led to possible errors in data recording or measurement. The number of patients with *exon 21 L858R* point mutation was disproportionately small. Only about half of the patients with PD were investigated for acquired resistance which was limited to *T790 M* mutation and histologic transformation. Fatigue is a subjective symptom which could have been underreported by the patients during clinic visits.

## Conclusions

Afatinib is an effective first-line treatment for patients with *EGFR*-mutant NSCLC. It is associated with good response rate and prolonged PFS. Patients with unfavorable clinical characteristics such as rare or complex *EGFR* mutations, symptomatic brain metastases, poor ECOG performance status, and inadequate organ function also benefit from first-line afatinib treatment. The side-effects of afatinib are moderate and *T790 M* mutation is the most common resistance mechanism identified.

## Data Availability

The datasets used and/or analyzed during the current study are available from the corresponding author on reasonable request.

## Abbreviations

CI: Confidence interval
CT: Computed tomography
CTCAE v4.0: Common Terminology Criteria for Adverse Events version 4
DCR: Disease control rate
ECOG: Eastern Cooperative Oncology Group
*EGFR*: *Epidermal growth factor receptor*
mOS: Median overall survival
mPFS: Median progression-free survivals
NSCLC: Non-small cell lung cancer
OR: Odds ratio
ORR: Objective response rate
PCR: Polymerase chain reaction
SD: Standard deviation
TAP: Thorax, abdomen and pelvis
TKI: Tyrosine kinase inhibitor
